# Enhancement of Glossiness Perception by Retinal-Image Motion: Additional Effect of Head-Yoked Motion Parallax

**DOI:** 10.1371/journal.pone.0054549

**Published:** 2013-01-15

**Authors:** Yusuke Tani, Keisuke Araki, Takehiro Nagai, Kowa Koida, Shigeki Nakauchi, Michiteru Kitazaki

**Affiliations:** Department of Computer Science and Engineering, Toyohashi University of Technology, Toyohashi, Aichi, Japan; University of Sussex, United Kingdom

## Abstract

It has been argued that when an observer moves, a contingent retinal-image motion of a stimulus would strengthen the perceived glossiness. This would be attributed to the veridical perception of three-dimensional structure by motion parallax. However, it has not been investigated whether the effect of motion parallax is more than that of retinal-image motion of the stimulus. Using a magnitude estimation method, we examine in this paper whether cross-modal coordination of the stimulus change and the observer's motion (i.e., motion parallax) is essential or the retinal-image motion alone is sufficient for enhancing the perceived glossiness. Our data show that a retinal-image motion simulating motion parallax without head motion strengthened the perceived glossiness but that its effect was weaker than that of motion parallax with head motion. These results suggest the existence of an additional effect of the cross-modal coordination between vision and proprioception on glossiness perception. That is, motion parallax enhances the perception of glossiness, in addition to retinal-image motions of specular surfaces.

## Introduction

When light from the surface of an object registers on our retinae and stimulates the photoreceptors, we are able to see the object. Light from an object is typically either emitted or reflected light, with reflection being far more common in natural environments than emission. In terms of human vision, reflection, scattering, and penetration are the most important interactions.

Light reflected from a surface can be classified as specular reflection, in which the angle of the reflected light is equal to the angle of incidence (cf. the Phong reflection model [1]), and diffuse reflection, in which light is reflected in almost all directions isotropically. The reflection spectrum can be approximated as the sum of the specular and diffuse components (cf. the dichromatic reflection model [2]), and the perceived intensity of the specular component depends on the position of the light source relative to the surface, the intensity of the light, the characteristics of the surface, and the position of the observer. Therefore, the specular component is often localized. In contrast, the diffuse component is often broadly distributed. The perceived intensity of the diffuse component does not depend on the viewing position but does depend on the incident angle (that is, the intensity is proportional to the cosine of the angle; cf. Lambert's cosine law). If the light source is a point source, the perceived intensity of the diffuse component also depends on the distance between the light source and the surface because the intensity of the radiation is inversely proportional to the square of the distance. Generally, the specular component mainly contributes to the highlights, glossiness, and ambient reflection, while the diffuse component contributes to the color of the object because the spectrum of the diffuse component is influenced by the spectral characteristics of the surface. On the other hand, the spectrum of the specular component is independent of the surface characteristics, and it is the same as that of the incident light (cf. NIR assumption [3]).

In computer graphics, the reflection, scattering, and penetration components can be rendered separately in each scene, but in our visual system, it seems impossible to achieve this kind of classification. The specular reflection component is either partially or fully polarized. Our visual system can vary the response according to the state of polarization (“Haidinger's brush” [4]), but this is a limited case and it seems to have no role in visual function (but see [5]). Incidentally, it is known that some arthropods (such as beetles and mantis shrimp) and fish can perceive and make use of the polarization of light [6]. The position of the light source, the form of the object, the surface reflectance, and so on are all known parameters in computer graphics and can be precisely defined, but the role of our visual system is to resolve such properties from the results of their interactions. This is known as “inverse optics” and constitutes an “ill-posed” problem [7]. A number of studies about the “shape from shading” have argued that the spatial structure of the light distribution depends on the relationship between viewing position, object, and light source, and that the human visual system would make use of this structure to perceive the surface orientation or curvature [8], [9]. Nevertheless, it seems as if our visual perception is constructed by using these components separately; namely, the glossiness is determined from the specular reflectance, the color from the diffuse reflectance [10], and the translucency from sub-surface scattering. For example, the perceived orientation of the surface was more precise with a specular surface than with an asperity surface [11], but was not affected by specular highlights and cast shadows [12], [13], nor by the strength of specularity [11]. These suggest that the specular components should be distinguished from the diffuse components in the human visual system [9], and physiological evidence has been presented that there are macaque neurons or cortical areas that possess selectivity for glossiness [14], [15], but how the human visual system achieves this is still unknown [16].

The perceived glossiness is affected by the object shape or orientation of the surface [17]–[20]. Contrary reports have stated that image features or image statistics derived from earlier visual processing are possibly related to glossiness [21] and translucency [22] perception. For example, a correlation between the skewness of the luminance histogram of the image and the intensity of the perceived glossiness was found [21]. However, the importance of the spatial structure of the light distribution, which results from the shape of the object, the illumination field, and their interaction with glossiness perception, was reemphasized by many studies [16], [23]–[32].

Binocular disparity between the specular highlight and the surface also affects the perceived glossiness. It has been reported that an appropriate disparity enhances the realism or authenticity of the perceived glossiness [33]–[38].

In our daily life, we rarely misperceive the reflectance property of a surface even when distortions such as splashes of mud or paint stains are present. One possible reason for this is that our visual system can obtain multiple visual images from different viewpoints sequentially, and we use these to estimate the surface properties. The relationship between the light source, object, and viewpoint is dynamic in reality. The retinal-image motion of specular reflection, the “specular flow,” differs from that of diffuse reflection [8], [39]–[43]. This difference can affect the perceived specularity, shininess, and glossiness of the surface [37]–[38], [40]. Although specular flow shown by a random-dot kinematogram cannot be perceived as shiny [41], specular flow or image velocity conveys some important information about the surface, especially the existence of the specular reflection.

Both the object motion and viewpoint motion induce retinal-image motion of the object. In static viewing, simulated versions of both types of retinal-image motion have similar effects on the glossiness matching performance or “glossiness constancy” [19], [34]. Human observers utilize retinal-image motions, which are the results of self-motion, to perceive various object and surface properties [44], [45]. The human visual system interprets some retinal-image motions as having been generated by self-motion if the retinal-image motions are spatially congruent with and temporally yoked by self-motion. Such a veridical relationship is needed for motion parallax. Sakano and Ando [38] examined the effects of the combination of self-motion and contingent retinal-image motion (i.e., motion parallax) on perceived glossiness; the conditions they employed are summarized in Table 1. They showed that when the observer moved his or her head and the stimulus changed correspondingly, a stronger glossiness was perceived than when both the observer and stimulus were static. They also showed that when there was no temporal change in the stimulus, the observer's motion did not affect the intensity of the perceived glossiness (i.e., head motion itself had no effect). However, the effect of the retinal-image motion itself, which simulated self-motion, was not investigated. Although Hartung and Kersten [40] and Doerschner et al. [43] have demonstrated the striking effect of retinal-image motion on the perceived shininess, the retinal-image motions they used were not the result of viewpoint motion, but of the object motion. Thus, it has not been investigated whether the effect of motion parallax is more than that of retinal-image motion itself.

**Table 1:** Conditions employed by Sakano and Ando (2010).

	Stimulus condition	
Viewing condition	Dynamic	Static
Dynamic	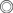	○
Static		○

The double circle indicates that the effect on glossiness perception was stronger.

In static viewing, a retinal-image motion indicates either a change in the lighting conditions or a change in the object, or both. It has been shown that we can distinguish the local reflectance change from a change in the position of the light source [46], [47]. If the effect of stimulus change as shown by Sakano and Ando [38] is the result of motion parallax and enhancement of the perception of the three-dimensional structure of the object, a stimulus change without any motion of the observer, i.e., visually simulating motion parallax in static viewing, would have a weaker effect than in dynamic viewing. Alternatively, if the cross-modal coordination of the retinal-image motion of the stimulus (vision) and the observer's self-motion (proprioception and/or motor command) is not crucial and observations under multiple lighting conditions are sufficient (cf. for lightness perception [48]), then a retinal-image motion that visually simulates motion parallax would enhance the intensity of perceived glossiness equally, irrespective of the observer's motion. For depth perception, it is known that the retinal-image motion visually simulating motion parallax brings about a weaker impression of depth in static viewing than in dynamic viewing [49].

In this paper, we investigate the effects of motion parallax in static viewing on glossiness perception compared with the corresponding effects in dynamic viewing by varying the intensity of the specular reflectance. In other words, the purpose of this paper is to examine whether cross-modal coordination of the retinal-image motion and observer's motion (motion parallax with observer's self-motion) is more effective than the retinal-image motion or whether the retinal-image motion alone is sufficient for the enhancement of perceived glossiness.

## Materials and Methods

### Participants

Nine undergraduate and graduate students participated in the experiment. All participants had normal or corrected-to-normal visual acuity and were unaware of the purpose of the experiment at the time of volunteering. After the experimental procedure was explained, they gave informed consent to participate in writing before the experiment began. The study was approved by the Committee for Human-Subject Studies at Toyohashi University of Technology.

### Apparatus

We presented the stimuli on a 22-inch cathode ray tube (CRT) monitor (40.0 cm ×30.0 cm, Mitsubishi RDF223H, 800×600 resolution, refresh rate of 120 Hz), and we ran the stimulus presentation, response acquisition, and response recording program on a desktop computer (Dell Vostro Desktop 220). Head positions were monitored during the experiment using an electromagnetic motion tracking system (Polhemous FASTRAK) with a data acquisition frequency (120 Hz) and transfer time (3.6 ms at 57,600 bps) that allowed us to synchronize the stimulus with head motion in real time.

### Stimuli

**Figure 1:**
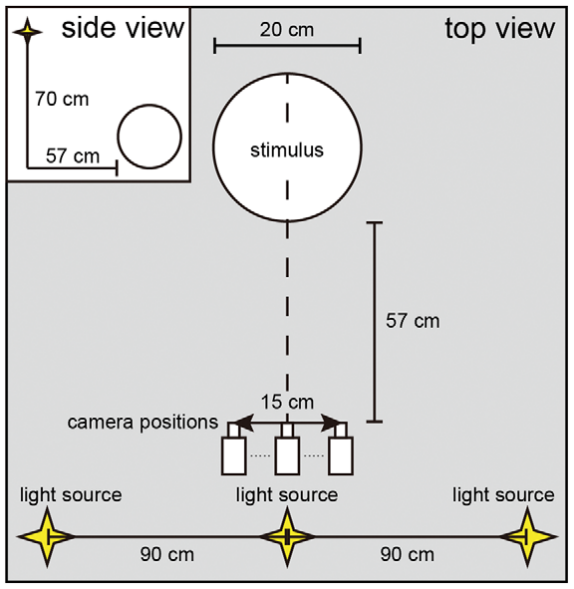
Relationship between the viewpoints (camera positions), the stimulus, and the light sources. The stimulus was illuminated by three fixed point-source lights. The distance between the stimulus and the camera positions was 57 cm, the same as the distance between the monitor and participant.

**Figure 2:**
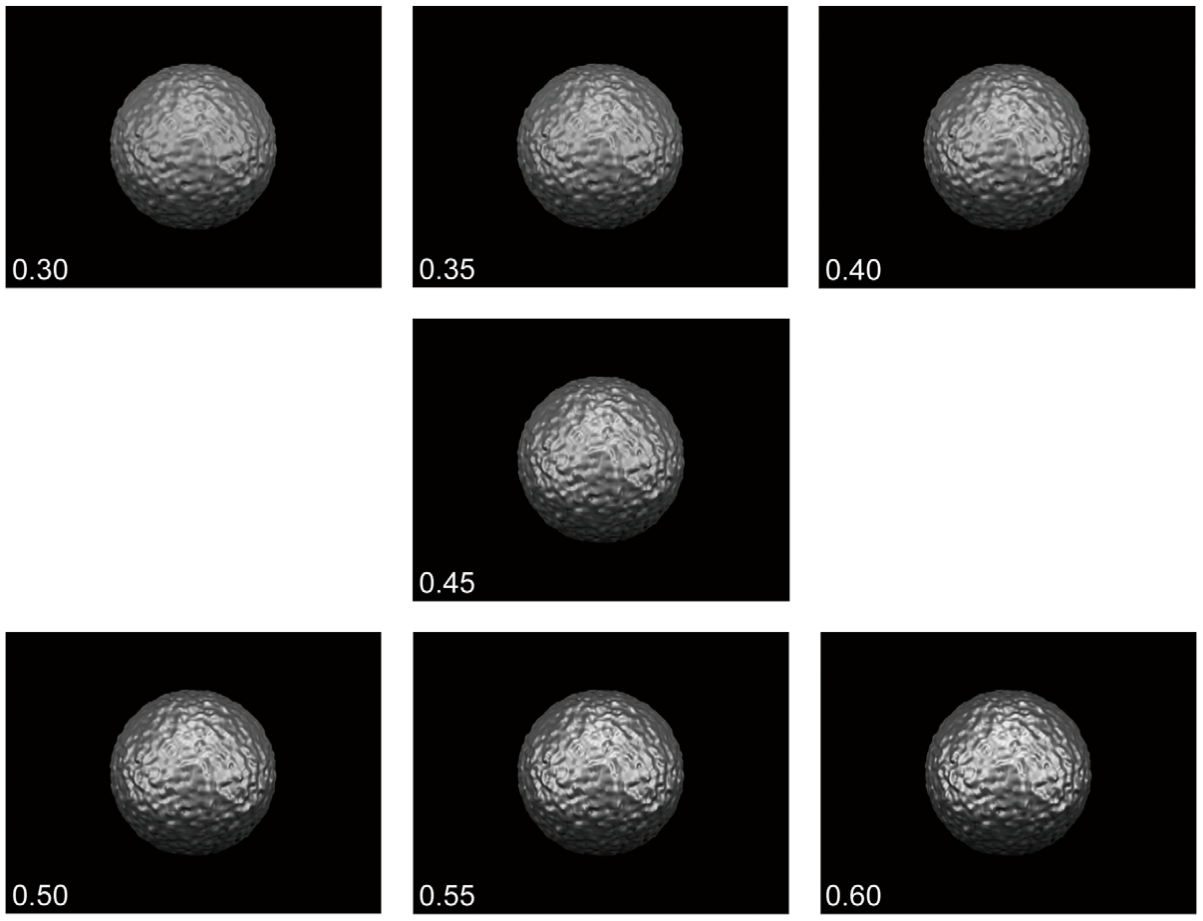
Examples of the stimuli. The stimuli were computer-generated spherical achromatic objects that were glossy and bumpy. They were modeled and rendered using POV-Ray (ver. 3.6.2). Stimuli for seven specular reflectance levels are shown: 0.30, 0.35, 0.40, 0.45, 0.50, 0.55, and 0.60. The diffuse reflectance was set so that the mean luminance of all stimuli was equal.

The stimuli, computer-generated spherical achromatic objects that were glossy and bumpy, were modeled and rendered using POV-Ray (ver. 3.6.2). In the virtual space, the diameter of each stimulus was 20 cm, and the viewing distance was 57 cm. The distance from the monitor to the observer was also 57 cm, and so the stimulus subtended an angle of 17.0°. The viewing points were set at intervals of 0.1 cm within a span of 15 cm. A total of 151 images from each viewing point were generated for each stimulus motion. The images were presented sequentially and cyclically at a frequency of 0.25 Hz with a sinusoidal speed change, which is described in the following paragraphs. The three light sources were fixed (Figure 1). We set seven levels of specular reflectance and diffuse reflectance to keep the mean luminance of all stimuli equal (Figure 2). These levels of specular reflectance were arbitrarily chosen by subjective observation and preliminary experiments.

### Procedure

All experiments were conducted in a darkened room. The independent parameters were the viewing condition, stimulus condition, and strength of the specular reflectance; all were within-subject parameters. For the viewing condition, the participants either observed the stimulus by moving their head to the left and right by 15 cm at 0.25 Hz (*D*_observer_: dynamic observer) or without moving (*S*_observer_: static observer). The retinal image of the stimulus either changed sequentially (*D*_image_: with retinal motion) or did not change (*S*_image_: without retinal motion) during observation. Hereafter, we describe these four conditions as *D*_image_-*D*_observer_, *D*_image_-*S*_observer_, *S*_image_-*D*_observer_, and *S*_image_-*S*_observer_ (stimulus condition-viewing condition). The *D*_image_-*D*_observer_ condition is a valid case of motion parallax with the observer's head motion where there is a stationary object in an environment.

The participants underwent head-motion training before the experiments to ensure appropriate travel distance, timing, and speed of their head motion. In the training session, we presented a marker that automatically moved at the ideal speed and travel length (0.25 Hz, 15 cm) in addition to another marker that tracked the observer's head motion. Participants tried to match these markers by swaying their upper body for a duration of 30–60 min (where the duration depended on the participant's performance and willingness to continue).

The strength of the specular reflectance was changed from 0.30 to 0.60 in steps of 0.05, resulting in 28 comparison conditions. The trials were divided into 16 sessions, during which the participants observed the stimulus monocularly. Eight sessions were conducted for each eye. Each session involved all stimuli conditions, and 28 trials were undertaken; each participant completed 448 trials. The participants also each wore a helmet with a magnetic probe to monitor their head position.

**Figure 3:**
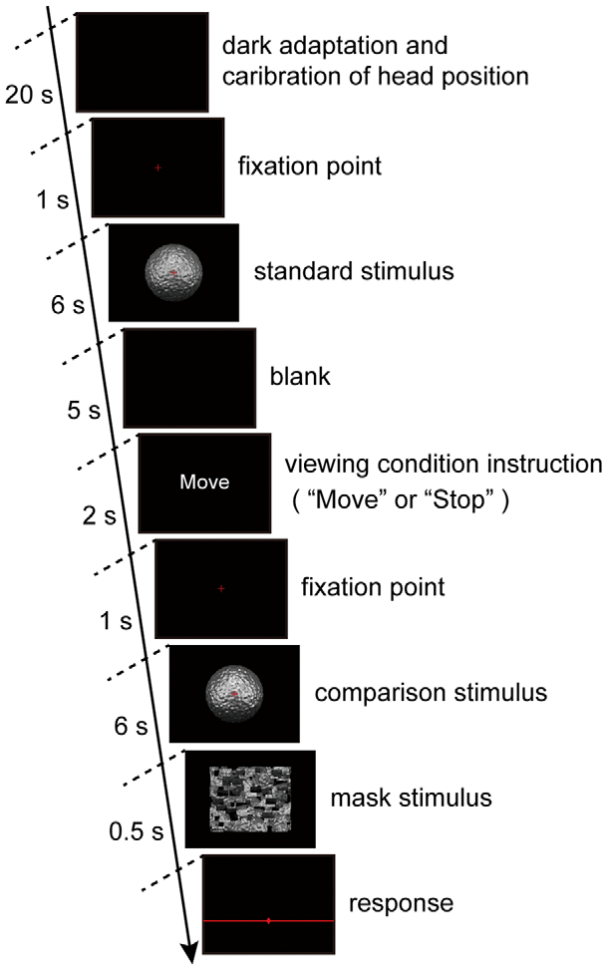
Flowchart of the experimental procedure. The time sequence of one session is shown. The first three frames were presented once and then those from the blank to the response were repeated 28 times. Further details are given in the text.

The task was to rate the relative strength of the perceived glossiness of the comparison condition to the standard condition using magnitude estimation. Our procedure resembled that followed by Sakano and Ando [38]. Whereas their participants provided a response in the form of a number, our participants responded by indicating a position along a line segment like as VAS (visual analogue scale) as described below. The aim of this approach was to elicit a direct value representing the participants' intuitive perception of the glossiness. The standard condition was a static stimulus with a specular reflectance of 0.45, which was in the middle of the strength levels we employed; the participant observed this in the *S*_image_-*S*_observer_ condition. A session was executed as follows: Calibration of the head position was performed first. After the fixation point was presented, the participant observed the standard stimulus for six seconds and was instructed to remember the strength of the glossiness; the standard condition was presented only once in each session. Five seconds after the standard stimulus disappeared, an instruction about the viewing condition was presented on the display for one second. The participant obeyed this instruction and observed the comparison stimulus for six seconds followed by a mask (jumbled stimuli) presented for 0.5 s. The participant then indicated the strength of the perceived glossiness of the comparison stimulus by using a mouse to click on a horizontal line displayed on the monitor (Figure 3). The center of the line corresponded to the glossiness strength of the standard condition, positions to the left meant that the glossiness of the comparison stimulus was weaker, and those to the right meant that the glossiness was stronger. The magnitude of the distance from the center indicated the relative difference. After this, the participant returned their head to the initial position for the next trial.

## Results

**Figure 4:**
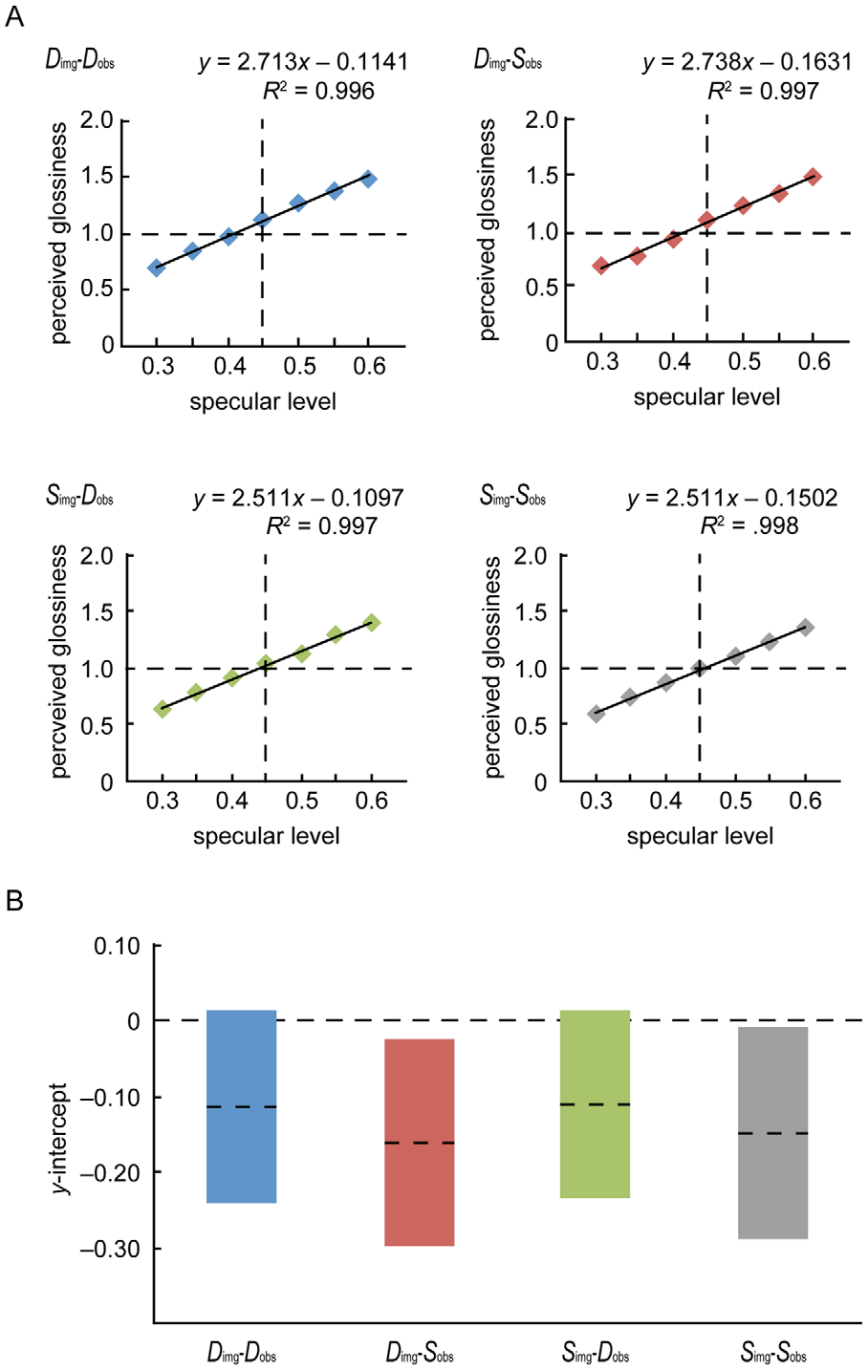
Perceived glossiness as a function of specular level for the four conditions. A. The solid lines show the results of the linear regression of the data in the *D*_image_-*D*_observer_, *D*_image_-*S*_observer_, *S*_image_-*D*_observer_, and *S*_image_-*S*_observer_ conditions. The data are well fitted in all conditions. The glossiness of the standard condition [specular level 0.45 in Fig. 2.] was defined to be exactly 1.0. B. The values of the *y*-intercept of each regression line. The dashed lines represent the average value, and the length of each bar represents the standard error of the mean.

To convert the selected line positions into numerical data, the extreme left of the line was set to 0.0, the center of the line to 1.0, and the right to 2.0. Since there was no constant error caused by the order of the sessions or by the participants, average values for each condition across sessions and participants were calculated and plotted as a function of the specular level. We applied a linear regression analysis, which revealed that the four functions could be regarded as linear (*R* ^2^>0.99), and the *Y*-intercept values of the regression lines did not vary significantly from 0 (Figure 4). This confirmed that our procedure is appropriate for representing perceived glossiness strengths and that the glossiness strength is dependent on the specular reflectance level even though the mean luminance of objects was constant [18]. The strength dependence was also supported by a three-way repeated-measures ANOVA that revealed significant main effects of all variables and the interaction between the specular level and stimulus condition (specular level: *F*(6, 48) = 74.529, *p*<0.00001, *η_p_^2^* = 0.903; stimulus condition: *F*(1, 8) = 14.385, *p* = 0.0053, *η_p_^2^* = 0.643; viewing condition: *F*(1, 8) = 10.434, *p* = 0.0121, *η_p_^2^* = 0.566; interaction: *F*(6, 48) = 3.314, *p* = 0.0082, *η_p_^2^* = 0.293).

To compare the perceived glossiness strengths, the results in the *S*_image_-*S*_observer_ condition were regarded as a baseline, and we calculated the ratios *D*_image_-*D*_observer_/*S*_image_-S_observer_, *D*_image_-S_observer_/*S*_image_-S_observer_, and *S*_image_-*D*_observer_/S_image_-*S*_observer_. A two-way repeated-measures ANOVA showed a significant main effect of the condition (*F*(2, 16) = 5.195, *p* = 0.0183, *η_p_^2^* = 0.394) and significant interaction between the condition and specular level (*F*(12, 96) = 2.248, *p* = 0.0150, *η_p_^2^* = 0.219). Post-hoc multiple comparisons for the main effect of the condition, the difference between the *D*_image_-*D*_observer_ condition and the *S*_image_-*D*_observer_ condition was significant (*p* = 0.005, *d* = 0.619), and other differences were not significant (*D*_image_-*D*_observer_ vs. *D*_image_-*S*_observer_: *p* = 0.102, *d* = 0.342, *D*_image_-*S*_observer_ vs. *S*_image_-*D*_observer_: *p* = 0.156, *d* = 0.330).

**Figure 5:**
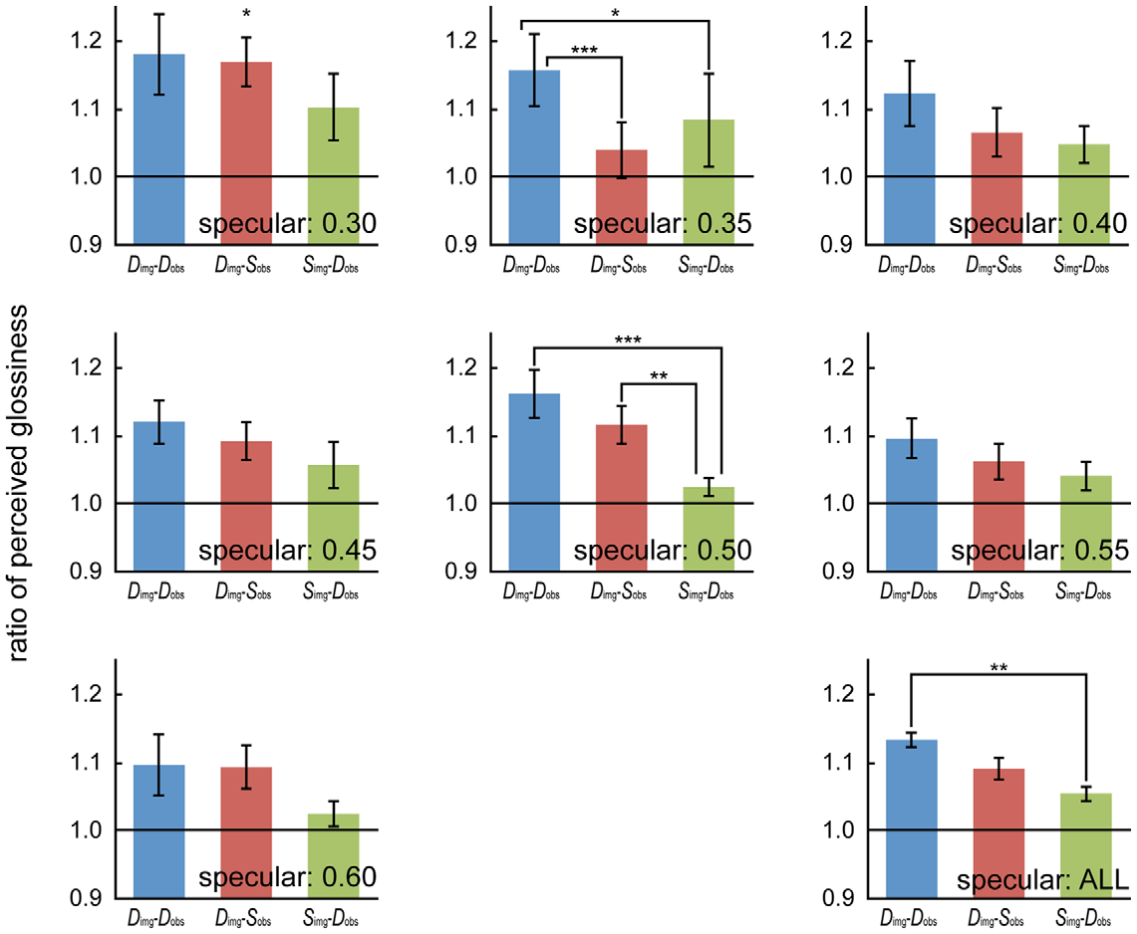
Comparison of the results for each condition. The perceived glossiness ratios of the *D*_image_-*D*_observer_, *D*_image_-*S*_observer_, and *S*_image_-*D*_observer_ conditions to the *S*_image_-*S*_observer_ condition for specular levels between 0.30 and 0.60 and for all specular levels. The error bars represent the standard error of the mean. The asterisks indicate the existence of a significant difference [*: *p*<0.05, **: *p*<0.01; ***: *p*<0.001].

The simple main effects of the condition were significant at specular levels of 0.30 (*F*(2,112) = 3.163, *p* = 0.0461), 0.35 (*F*(2,112) = 6.178, *p* = 0.0028), and 0.50 (*F*(2,112) = 8.635, *p* = 0.0003). In multiple comparisons for a specular level of 0.35, the perceived glossiness in the *D*_image_-*D*_observer_ condition was stronger than those in the *D*_image_-*S*_observer_ (*p* = 0.0007, *d* = 0.818) and *S*_image_-*D*_observer_ (*p* = 0.0329, *d* = 0.393) conditions. The difference between the *D*_image_-*S*_observer_ condition and the *S*_image_-*D*_observer_ condition was not significant (*p* = 0.1892, *d* = 0.259). For a specular level of 0.50, the perceived glossiness in the *S*_image_-*D*_observer_ condition was weaker than those in the *D*_image_-*D*_observer_ (*p* = 0.0001, *d* = 1.695) and *D*_image_-*S*_observer_ (*p* = 0.0074, *d* = 1.351) conditions. The difference between the *D*_image_-*D*_observer_ condition and the *D*_image_-*S*_observer_ condition was not significant (*p* = 0.1804, *d* = 0.471). No significant difference was observed for a specular level of 0.30 in post-hoc multiple comparisons, but we obtained medium-size effects for the superiority of *D*_image_-*D*_observer_ over *S*_image_-*D*_observer_ and that of *D*_image_-*S*_observer_ over *S*_image_-*D*_observer_ (*D*_image_-*D*_observer_ vs. *D*_image_-*S*_observer_: *p* = 0.7332, *d* = 0.078, *D*_image_-*D*_observer_ vs. *S*_image_-*D*_observer_: *p* = 0.0217, *d* = 0.480, *D*_image_-*S*_observer_ vs. *S*_image_-*D*_observer_: *p* = 0.049, *d* = 0.518). Furthermore, the means of the three ratios were significantly larger than 1. That is, the perceived strengths in the *D*_image_-*D*_observer_ (*t*(8) = 3.869, *p* = 0.005, *d* = 1.368), *D*_image_-*S*_observer_ (*t*(8) = 4.144, *p* = 0.003, *d* = 1.465), and *S*_image_-*D*_observer_ (*t*(8) = 2.935, *p* = 0.019, *d* = 1.038) conditions were stronger than that in the *S*_image_-*S*_observer_ condition (Figure 5).

## Discussion

Our data showed that the perceived glossiness in the *D*_image_-*D*_observer_ condition was stronger than that in the *S*_image_-*S*_observer_ condition. This means that valid motion parallax with head motion enhances the glossiness perception, which is consistent with the results of Sakano and Ando [38]. Since the perceived glossiness in the *D*_image_-*S*_observer_ condition was also stronger than that in the *S*_image_-*S*_observer_ condition, the retinal-image motion without head motion appears to strengthen the perceived glossiness and our visual system makes use of this multiple visual information to process glossiness, similarly to how shininess is processed [40], [43].

Moreover, the effect in the *D*_image_-*D*_observer_ condition was stronger than that in the *D*_image_-*S*_observer_ condition at the specular level of 0.35, and at any other specular levels, the effect in the *D*_image_-*D*_observer_ condition was not weaker than that in the *D*_image_-*S*_observer_ condition. We consider these results to demonstrate the existence of an additional cross-modal coordination effect between vision and proprioception (or motor command). The perception of three-dimensional structures via motion parallax with head motion could be considered to contribute to the enhancement of perceived glossiness in addition to just retinal-image motion. However, since the difference between the *D*_image_-*D*_observer_ and *D*_image_-*S*_observer_ conditions differed with the specular reflectance level, the effect of the cross-modal coordination may not be strong.

The perceived glossiness in the *S*_image_-*D*_observer_ condition was stronger than that in the *S*_image_-*S*_observer_ condition, which means that the observer's motion itself strengthens the perceived glossiness. This is inconsistent with the results of Sakano and Ando [38]; however, the effect in the *S*_image_-*D*_observer_ condition was generally weak, the difference between the *S*_image_-*D*_observer_ and *S*_image_-*S*_observer_ condition results varied with the specular reflectance level, and there were levels for which the effect in the *S*_image_-*D*_observer_ condition was very small (e.g., at the specular level of 0.50). This point should be investigated carefully in future studies.

One may argue that the stimulus (a bumpy sphere) and the surrounding environment (three lights against a black background) used in the experiment were unnatural and inadequate for investigating human natural vision. However, this is a first step to examining the effects of the cross-modal coordination on glossiness perception. We would like to conduct research with experiments in natural environments, which are constructed using an illumination map (e.g. Debevec's database [50]). Another possible concern about the experimental methods is with regard to the rating task. Participants could have different subjective criteria for judging the relative difference, and the criterion used by a participant might change in the course of the experiment. First, we checked the variance and sequential change of each participant's responses across sessions. There was not any constant tendency. One might think that in the initial trial, before seeing the stimuli in the rest of the trials, participants could have difficulties in rating the first stimulus that they encountered, but this effect seemed small in this study. To cope with the difference of subjective criteria, we regarded the *S*_image_-*S*_observer_ condition as a baseline, and calculated the ratios *D*_image_-*D*_observer_/*S*_image_-*S*_observer_, *D*_image_-*S*_observer_/*S*_image_-*S*_observer_, and *S*_image_-*D*_observer_/*S*_image_-*S*_observer_ for the analysis. Moreover, we employed a repeated-measures ANOVA that statistically considers individual differences.

This paper has presented the first report on the separate effects of retinal-image motion and motion parallax on the perception of glossiness in a quantitative manner. We have suggested here that the retinal-image motion over specular surfaces enhances the perception of glossiness, and additionally the cross-modal coordination of the retinal-image motion and self-motion as motion parallax also contribute. However, the effect of the retinal motion and the effect of motion parallax were different for specular levels. We have no explicit explanation for this variance, but it might be attributed to individual differences in quantitative specular sensitivity since the qualitative tendency was constant for almost all specular levels: The effect of motion parallax with head motion was found to be greater than the effect of retinal image motion, which was greater than the effect of a stationary image. To control this variance, our future work will employ individual optimization of specular levels.
